# PREVALENCE AND HERITABILITY OF PERCEIVED MENTAL FATIGABILITY IN THE LONG LIFE FAMILY STUDY

**DOI:** 10.1093/geroni/igz038.865

**Published:** 2019-11-08

**Authors:** Alexa J Meinhardt, Theresa Gmelin, Allison L Kuipers, Stacy L Andersen, Stephanie Cosentino, Mary K Wojczynski, Kaare Christensen, Nancy W Glynn

**Affiliations:** 1 University of Pittsburgh, Department of Epidemiology, Pittsburgh, Pennsylvania, United States; 2 University of Pittsburgh Graduate School of Public Health, Pittsburgh, Pennsylvania, United States; 3 Boston University School of Medicine, Boston, Massachusetts, United States; 4 Columbia University Department of Neurology, New York, New York, United States; 5 Washington University School of Medicine in St. Louis Department of Genetics, St. Louis, Missouri, United States; 6 University of Southern Denmark, Odense, Nordjylland, Denmark

## Abstract

We examined the prevalence and heritability of perceived mental fatigability among older adults enrolled in the Long Life Family Study. Participants (N=2342; 55% female) self-administered the Pittsburgh Fatigability Scale (PFS; scores range 0-50; higher score=greater fatigability). Using the PFS mental subscale, we evaluated differences across age strata (adjusted for family structure and field center) and estimated genetic heritability using the variance covariance methods implemented in SOLAR to determine genetic heritability (adjusted for age, sex, and field center). PFS mental score (mean±SD) and prevalence of higher mental fatigability (PFS ≥13) was greater across age strata: 60-69 (N=996, 5.9± 6.5, 14.5%), 70-79 (N=830, 6.8 ±7.6, 18.7%), 80-89 (N=251, 11.7±10.8, 41.8%), and ≥90 (N=265, 20.2±13.6, 67.2%), p<0.0001. Only among those ≥90, females (21.7±13.5) had greater mental fatigability than males (18.0±13.5), p=0.03. Residual heritability of mental fatigability was 0.17, p<0.0001. Future analyses will evaluate correlates of mental fatigability to identify potential avenues for intervention.

